# New and Potent Quinuclidine-Based Antimicrobial Agents

**DOI:** 10.3390/molecules24142675

**Published:** 2019-07-23

**Authors:** Andreja Radman Kastelic, Renata Odžak, Iskra Pezdirc, Karlo Sović, Tomica Hrenar, Ana Čipak Gašparović, Mirjana Skočibušić, Ines Primožič

**Affiliations:** 1 Department of Chemistry, Faculty of Science, University of Zagreb, Horvatovac 102a, HR-10000 Zagreb, Croatia; 2 Department of Chemistry, Faculty of Science, University of Split, R. Boškovića 33, HR-21000 Split, Croatia; 3 KB “Sestre milosrdnice”, Vinogradska cesta 29, HR-10000 Zagreb, Croatia; 4 Institut Ruđer Bošković, Bijenička cesta 54, HR-10000 Zagreb, Croatia; 5 Department of Biology, Faculty of Science, University of Split, R. Boškovića 33, HR-21000 Split, Croatia

**Keywords:** antimicrobial potency, quinuclidinium oximes, gram-positive and gram-negative bacteria, multi-way analysis

## Abstract

Developing new antibiotics is currently very important since antibiotic resistance is one of the biggest problems of global health today. In the search for a new class of potential antimicrobial agents, ten new compounds were designed and synthesized based on the quinuclidinium heterocyclic core and the oxime functional group. The antimicrobial activity was assessed against a panel of representative gram-positive and gram-negative bacteria. All compounds demonstrated potent activity against the tested microorganisms, with the minimum inhibitory concentration (MIC) values ranging from 0.25 to 256.00 μg/mL. Among the tested compounds, two quaternary compounds, *para*-*N*-chlorobenzyl and *meta*-*N*-bromobenzyl quinuclidinium oximes, displayed the most potent and broad-spectrum activity against both gram-positive and gram-negative bacterial strains (MIC values from 0.25 to 4.00 μg/mL), with the lowest value for the important multidrug resistant gram-negative pathogen *Pseudomonas aeruginosa*. In the case of *Klebsiella pneumoniae*, activity of those compounds are 256-fold and 16-fold better than gentamicin, respectively. MTT assays showed that compounds are nontoxic for human cell lines. Multi-way analysis was used to separately reduce dimensionality of quantum chemical data and biological activity data to obtain a regression model and the required parameters for the enhancement of biological activity.

## 1. Introduction

Infectious diseases are the second leading cause of death worldwide and cause significant morbidity, having a profound effect on global health [1]. The emergence of multidrug-resistant bacteria has become a serious threat to public health and is considered one of the greatest challenges for contemporary medicine. The ESKAPE group of bacterial pathogens (*Enterococcus faecium*, *Staphylococcus aureus*, *Klebsiella pneumoniae*, *Acinetobacter baumannii*, *Pseudomonas aeruginosa*, and *Enterobacter species*) is responsible for a majority of hospital-acquired infections and presents critical threats in nosocomial pathogenesis, transmission, and resistance [2,3]. Various health organizations have spoken about the urgent need to develop new antibiotics, especially against drug-resistant gram-negative ESKAPE bacilli. For instance, the World Health Organization (WHO) has raised its utmost concern about the post-antibiotic era where common infections and minor injuries may result in significant morbidity and mortality [4]. An important potential strategy to help combat the resistance problem involves the discovery and development of new active agents capable of partly or completely suppressing bacterial resistance mechanisms.

Heterocyclic compounds are the key motifs in a wide range of natural products such as vitamins, carbohydrates, and nucleic and amino acids, and medicinal chemistry often engages in stimulating such natural motifs. Heterocyclic compounds constitute a major portion of the currently approved drugs by the Food and Drug Administration (FDA), and over 80% of top small molecule drugs by US retail sales in 2010 contain at least one heterocyclic fragment in their structure [5]. Synthetic heterocycles have widespread therapeutic uses such as antibacterial, antifungal, antimycobacterial, trypanocidal, anti-HIV activity, antileishmanial agents, genotoxic, antitubercular, antimalarial, herbicidal, analgesic, anti-inflammatory, muscle relaxants, anticonvulsant, anticancer and lipid peroxidation inhibitor, hypnotics, antidepressant, antitumoral, and anthelmintic and insecticidal agents [6,7,8,9,10].

**Figure 1:**
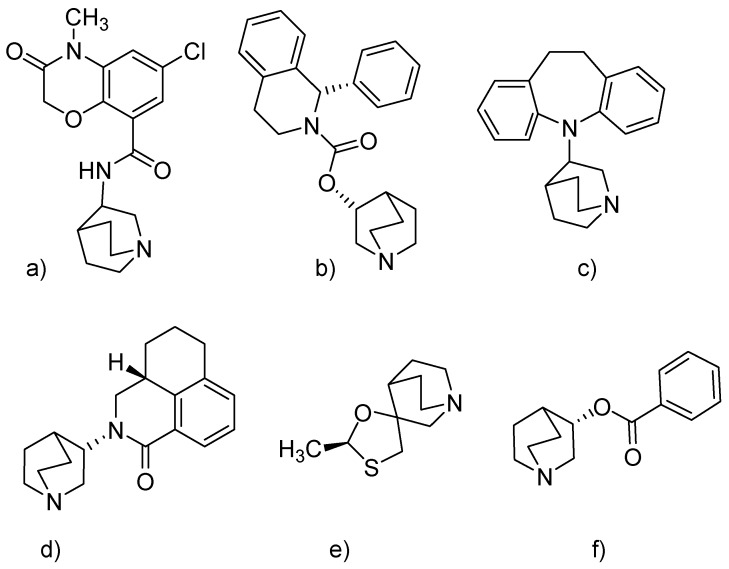
Quinuclidine-3-yl-based drugs: (**a**) Azasetron; (**b**) Solifenacin; (**c**) Quinupramine; (**d**) Palonosetron; (**e**) Cevimeline; and (**f**) Benzoclidine.

Quinuclidine, a bicyclic heterocyclic compound, is found in many natural products such as the bark of *Cinchona* trees and is isolated by alkaloid extraction [11]. It has also been used in the synthesis of catalysts which are useful in asymmetric reactions such as aldol reactions, Henry and Aza–Henry reactions, Diels–Alder reactions, etc. [12,13]. Quinuclidine moiety is used to prepare various therapeutically potent derivatives, such as FDA-approved drugs Azasetron, Benzoclidine, Palonosetron, Solifenacin, and Quinupramine, which contain Quinuclidine as its main component (Figure 1) [14].

Avibactam, the first non-β-lactam β-lactamase inhibitor, is also a quinuclidine-based drug, which showed excellent inhibitory activity against most class A and class C and some class D β-lactamases and is proven to inhibit penicillin-binding proteins. Thus, quinuclidines are recognized as a desirable structure and a useful template for design of novel compounds with potential pharmacological interest.

**Figure 2:**
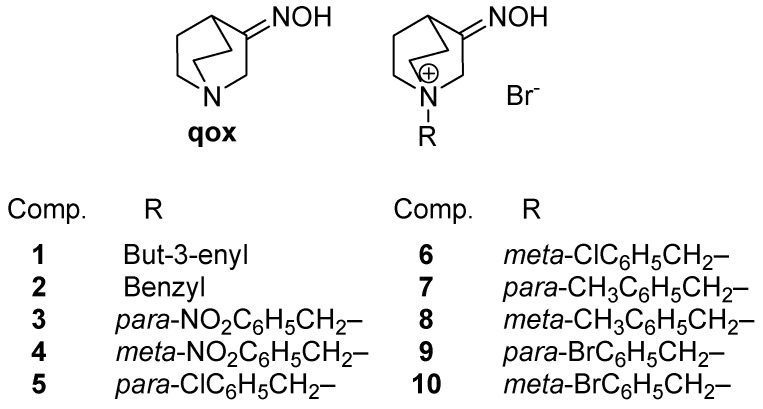
Synthesized compounds: 3-hydroxyiminoquinuclidine (**qox**) and quaternary derivatives **1**–**10**.

In our recent work, we reported synthesis of novel quaternary 3-hydroxy [15,16] and 3-hydroxyimino [17] quinuclidinium bromides with different alkyl chains lengths (3–16 carbon atoms) and showed that bicyclic quinuclidinium compunds have excellent and promising activity against most gram-positive and clinically relevant gram-negative bacterial strains compared to conventional antimicrobial agents. One of the expected antimicrobial mechanisms of cationic biocides involves the destructive interaction with the outer membrane [18]. In this work, ten different quaternary *N*-alkyl/alkylaryl derivatives of 3-hydroxyiminoquinuclidine were prepared; see Figure 2. All compounds are new and not previously described in the literature. For all compounds, antimicrobial activity toward multidrug-resistant gram-negative and gram-positive bacteria was estimated.

## 2. Results and Discussion

### 2.1. Synthesis of Compounds

**Figure 3:**
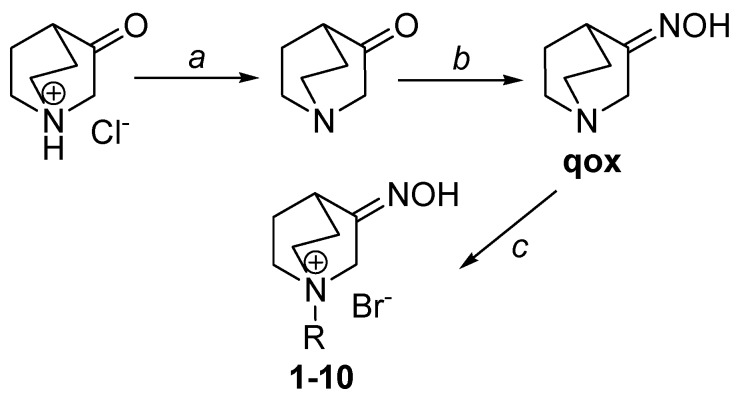
The synthetic route to target compounds. Reagents and conditions: (**a**) K_2_CO_3_, H_2_O, Et_2_O, 25 °C, 30 min; (**b**) NH_2_OH × HCl, NaOH, MeOH, 25 °C, 1 h; and (**c**) alkyl/aryl bromide, THF, Et_2_O, 66 °C, 5 h.

The initial compound for the oxime synthesis was quinuclidin-3-one prepared from commercially available quinuclidin-3-one hydrochloride by a previously described procedure with potassium carbonate as a base in a high yield [19]. 3-Hydroxyiminoquinuclidine was prepared by the reaction of quinuclidin-3-one with hydroxylamine hydrochloride and sodium hydroxide [20] (Figure 3). NMR analysis showed that prepared oxime has an (*E*)-configuration which is expected to be thermodynamically favored as determined previously [17]. To prepare compounds **1**–**10**, a Menshutkin reaction was employed to convert tertiary amine to quaternary ammonium salt by the reaction of appropriate alkyl/aryl halide. As a rule, the optimal conditions were obtained with 1 eq. of alkyl/aryl bromide in dry tetrahydrofurane at reflux temperature under nitrogen atmosphere. The products were obtained in 60–90% yield, and all of them were solids that precipitated from the reaction mixture using diethyl ether. The p*K*_a_ values for the oxime hydroxyl group and the quinuclidine nitrogen atom of qox were already determined experimentally: 10.805 ± 0.030 and 8.072 ± 0.034, respectively [17]. As expected, a positive charge on the quinuclidinium nitrogen atom upon quarternization stabilizes the conjugated base and the acidity of the oxime group of compound **10** is increased to 10.05 ± 0.03. Compounds **1**–**10** are new compounds and are not previously described in the literature.

### 2.2. Antimicrobial Activity

In this study, all synthesized novel *N*-alkyl derivatives of quinuclidinium oximes **1**–**10** were preliminary screened against a diverse panel of selected antibiotic susceptible gram-positive bacteria purchased from the American Type Culture Collection (ATCC), Rockville, MD, USA: *Bacillus cereus* ATCC 11778, *Enterococcus faecalis* ATCC 29212, *Staphylococcus aureus* ATCC 25923, and *Clostridium perfringens*; and antibiotic resistant gram-negative bacteria obtained from Microbiology Laboratory, Department of Biology, Faculty of Science, University of Split, Croatia (FNSST): *Escherichia coli* FNSST 982, *Klebsiella pneumoniae* FNSST 011, and *Pseudomonas aeruginosa* FNSST 982 by disc diffusion assay. Cefotaxime and gentamicin, which are well-known relevant antimicrobial agents [21,22] were included in the assays, serving as positive controls and as a basis for comparison. The compounds showed to be active against antibiotic susceptible gram-positive bacteria but also exhibited significant antibacterial effects against gram-negative gentamicin-resistant isolates. As shown in Table 1, the results of the disc diffusion assay pointed out that the compounds exhibit moderate to high potency and broad spectrum activity against the bacterial strains tested. The mean zones of inhibition of the target compounds against all bacterial strains tested were found in the range of 6.2 ± 1.3 to 29.8 ± 1.3 mm. It is noteworthy that, among the synthesized compounds, unsubstituted nonquaternary 3-hydroxyiminoquinuclidine (QOX) showed very poor activity against all strains tested (7.6 ± 1.1 to 10.8 ± 1.4 mm). The antimicrobial activity trend did not change significantly for quaternary compound **1** with butenyl group as a substituent, but the compounds containing benzyl moiety showed appreciably better antimicrobial effects; see Table 1. As expected, the strongest and most promising antimicrobial potential against a panel of tested strains was observed with compounds which have a halogen atom as a substituent (**5** and **6** with chlorine atoms at the *meta* and *para* positions of the benzyl substituent and **9** and **10** with bromine atoms). Among all tested derivatives, **10** showed the maximum inhibition against all tested bacterial strains (21.0 ± 0.8 to 29.8 ± 0.1.3 mm). The highest antibacterial activity was recorded against *Pseudomonas aeruginosa*, followed by *Klebsiella pneumoniae* and *Bacillus cereus*.

Antimicrobial efficacy was also evaluated by the broth microdilution method to determine the minimum inhibitory concentrations (MICs) of a series of novel derivatives of quinuclidinium oxime derivatives necessary to inhibit bacterial growth. Cefotaxime and gentamicin were also included in the assays as positive controls. The results presented in Table 2 show moderate to excellent antibacterial activity against tested strains with MIC values from 0.25 to 256.00 μg/mL. Nonquaternary derivative **qox** showed very poor activity against all strains tested (MIC = 256 μg/mL except for *S. aureus* and *P. aeruginosa* 128 μg/mL). On the other hand, evaluations pointed out compound **10** as the most active compound in the series, especially against gram-negative bacteria (MIC values from 0.25 to 1.00 μg/mL). Compound **10** exhibited excellent in vitro activity against a broad spectrum of clinically important resistant gram-negative pathogens such as *Pseudomonas aeruginosa* (MIC = 0.25 μg/mL) and *Klebsiella pneumoniae* (MIC = 0.50 μg/mL). These MICs are 256-fold and 16-fold better than gentamicin, respectively. Compound **5** also showed very strong activity against all tested gram-negative bacteria, with MIC values from 0.25 to 1.00 μg/mL. Compounds **7** and **8** showed very weak antimicrobial properties among quaternary derivatives. These compounds have a methyl group as a substituent on an aromatic ring that donates electrons, unlike the electronegative nitro group and halogen atom present in other substituted compounds. The data indicate that, in general, the quaternary *N*-benzyl derivatives of quinuclidine oximes **2**–**10** are more potent and have broader antimicrobial activity spectrums compared to their uncharged analog **qox** and butyl derivative **1**. A comparison of *meta*- and *para*-substituted derivatives reveals that the position of the substituent at the benzyl moiety does not correlate with antimicrobial efficacy.

### 2.3. MTT Assay

**Figure 4:**
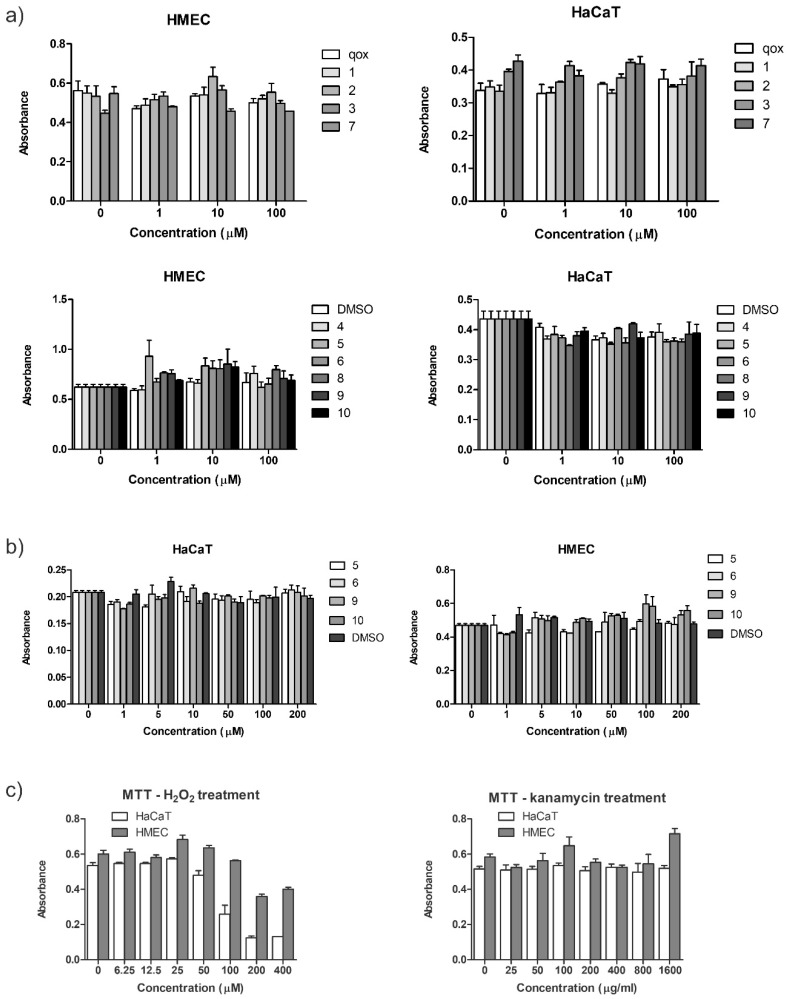
Effects of all synthesized compounds on normal human cell lines viability: Concentrations up to (**a**) 100 µM; (**b**) 200 µM; and (**c**) assay controls H_2_O_2_ and kanamycin.

The results of MTT assay are shown in Figure 4. Compared to normal physiological cell growth, the compounds did not cause significant changes in HaCat or HMEC cell growth; see Figure 4a. In addition, testing a wider concentration range of compounds with confirmed antibacterial activity showed no changes in cell viability; see Figure 4b.

### 2.4. Effects of the Compounds on Cellular Reactive Oxygen Species and Antioxidative Defence

**Figure 5:**
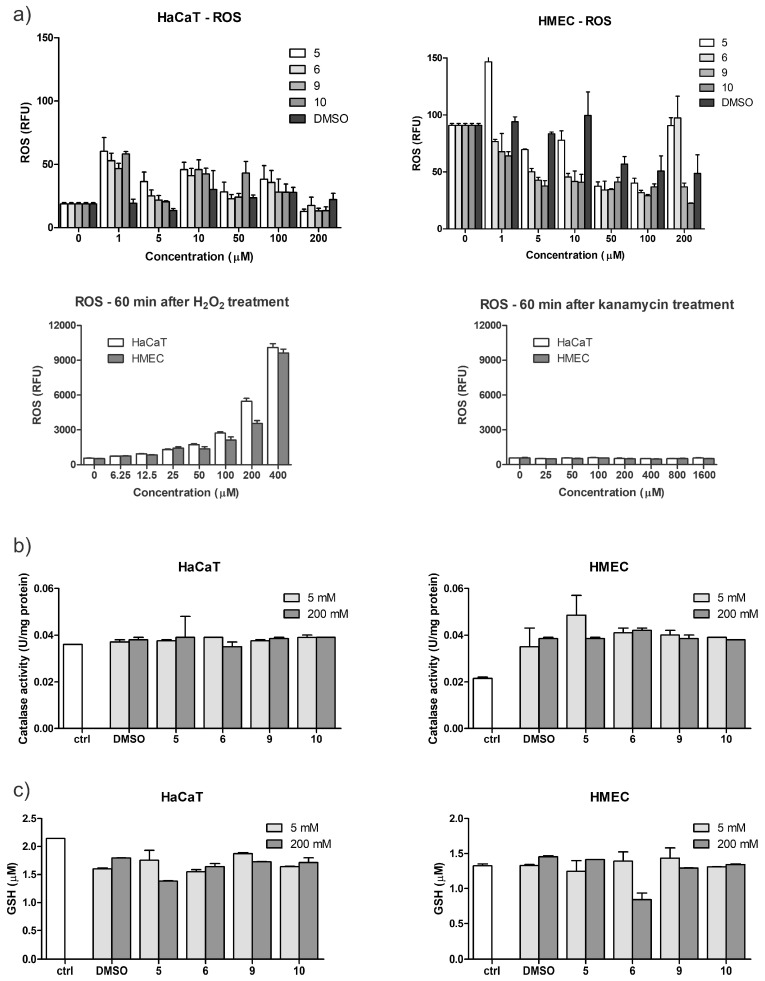
Tests of the ability of compounds **1**–**10** to change (**a**) intracellular ROS levels with assay controls H_2_O_2_ and kanamycin; (**b**) cellular antioxidative defense system GSH; and (**c**) catalase.

The effects of the compounds with antibacterial activity, **5**, **6**, **9**, and **10**, were further tested for the ability to change intracellular reactive oxygen species (ROS) levels and on the cellular antioxidative defense system *glutathione* (GSH) and catalase (Figure 5). The HMEC cell line showed higher basal levels of ROS when compared to the HaCaT cell line (*p* < 0.05) (Figure 5a). The compounds had different effects on ROS level depending on the cell line. In HaCaT cells, **5**, **6**, **9**, and **10** increased ROS at the lowest concentration applied and the effect diminished with increasing concentration of the compound. In HMEC cells, the compounds decreased ROS with the exception of 1 µM of **5** (*p* < 0.05). These results are in accordance with catalase activity (Figure 5b). In HaCaT cells, catalase activity is changed neither by DMSO nor the compounds. In contrast, in HMEC cells, the activity increased with addition of DMSO and the compounds; therefore, the effects of the compounds themselves cannot be resolved, as their effect on catalase activity is the same as that of DMSO. Unlike catalase, GSH levels are not changed by the addition of DMSO, **5**, **6**, **9**, or **10**, with the exception of 200 µM of **6** in HMEC cells (Figure 5c). These results indicate that DMSO could cause a slight shift in the cellular redox balance but without influencing cell viability. The potential mechanism is to be investigated. 

### 2.5. Multi-Way Analysis

**Figure 6:**
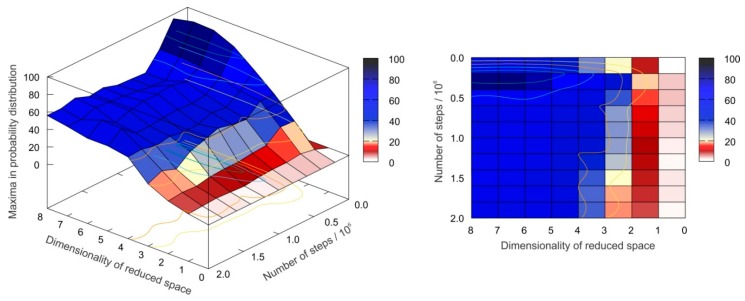
Total number of strict local maxima in the probability distribution for geometries of compound **2** in the reduced space calculated by the principal component analysis of the molecular dynamics trajectory.

To additionally improve the relationship between structure and antimicrobial activity, tensor decomposition methods were utilized on the potential energy surfaces (PES) and biological activity data. First, a full conformational analysis for all compounds was performed. Conformers were determined by using the recently developed approach consisting of ab initio molecular dynamics and probability search in reduced space [23]. The example of conducted conformational analysis is presented on Figure 6.

A plateau of the local maxima in probability distribution is achieved with 4 principal components and approx. 1,000,000 points in the molecular dynamics trajectory.

**Scheme 1:**
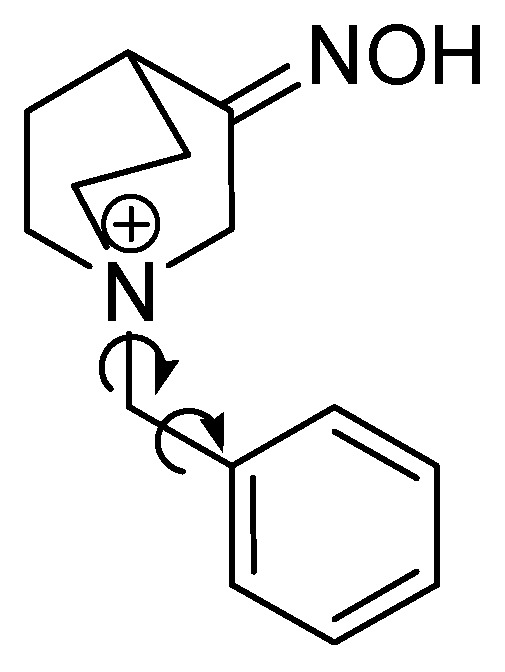
Torsional coordinates used in 2-D potential energy surface calculation.

The lowest energy conformer for each compound was subjected to quantum chemical calculation of two-dimensional PES, which was calculated in dependence on two torsional coordinates (Scheme 1).

**Figure 7:**
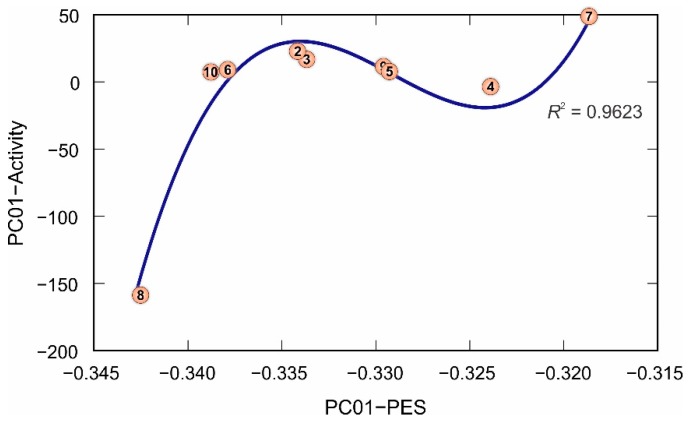
Multivariate regression model of the first principal component of biological activity in dependence on the first principal component of potential energy surfaces.

Equivalent torsional coordinates were used for all other compounds. After the 2-D PES scan, a multi-way analysis of energy data was performed for compounds **2**–**10**. Tensor of PES data was decomposed using the Tucker3 model, and the first principal component described 91% of the total variance among the data, providing an excellent description of molecular energetics. On the other hand, biological activity data were decomposed using principal component analysis (PCA) and the first principal component described 82% of the total variance among the data. Again, this one component was sufficient to describe all important trends in biological activity data. A polynomial regression model between the biological activity and potential energy surfaces was established and is presented on Figure 7.

**Figure 8:**
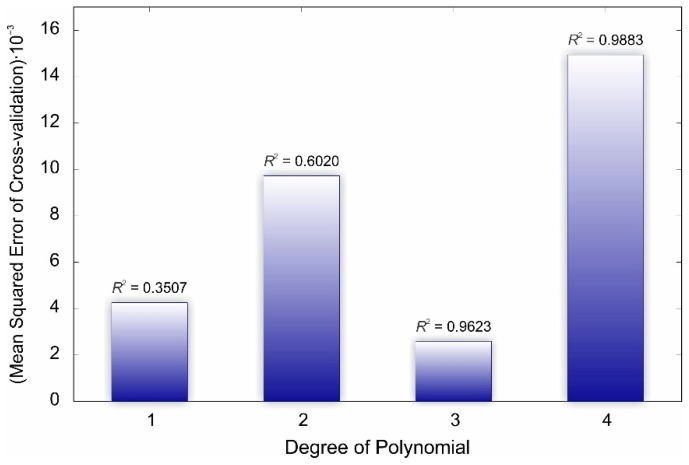
Mean squared error of model prediction obtained by leave-one-out cross validation on the set of the first principal component of biological activity in dependence on the first principal component of potential energy surfaces. (*R*^2^ values for each model are placed above the bars.)

The *R*^2^ value for the established polynomial model of degree 3 was 0.9623, and the model very well describes the biological activity of investigated compounds in relation to their potential energy surfaces. It can be seen that the two most active compounds **5** and **10** have almost the same value along the first principal component describing biological activity. In a similar matter, the two least active derivatives **7** and **8** present extrema points on the activity axis. A regression model between calculated potential energy surfaces and biological activity enables in silico experiments with compounds having slightly different variations in the structure. In this way, the structure of compounds can be adjusted and optimized in search of biological activity enhancement within the tested bacteria set. To confirm the validity of the established model, a leave-one-out cross validation procedure was performed. The mean squared error of prediction in dependence on the polynomial degree is presented in Figure 8.

The presented polynomial model of degree 3 has the lowest mean squared error with high value for the coefficient of determination *R*^2^, thus ensuring good prediction properties in the domain ranging within the values of the first principal component of the decomposed potential energy surfaces.

## 3. Materials and Methods 

### 3.1. Synthesis of Compounds

All reagents and solvents were used as purchased from commercial suppliers without further purification. Thin-layer chromatography (TLC) was performed on Aluminum oxide 60 F_254_ plates (Sigma-Aldrich, St. Louis, MO, USA) and visualized under UV light (254 nm) or by iodine fumes. Melting points were determined on a Melting Point B-540 apparatus (Büchi Labortechnik GmbH, Essen, Germany) and are uncorrected. Elemental analysis (CHN) was performed on a Perkin Elmer 2400 Series II CHNS analyzer (PerkinElmer, Inc., Waltham, MA, USA), and the purity of all compounds was ≥99%. FTIR spectra were recorded as KBr pellets on a Perkin-Elmer Spectrum Two (PerkinElmer, Inc., Waltham, MA, USA), and the signals were reported in cm^−1^. NMR spectra were recorded on a Bruker Avance III HD 400 MHz/54 mm Ascend spectrometer (Bruker Corporation, Billerica, MA, USA) at 22 °C in CDCl_3_ and DMSO with tetramethylsilane as the internal standard. Chemical shifts are given in ppm downfield from tetramethylsilane as the internal standard, and coupling constants are given (*J*) in Hz. Splitting patterns are designated as s (singlet), d (doublet), dd (doublet of doublets), t (triplet), q (quartet), or m (multiplet). Anhydrous reactions were carried out in dried glassware under a nitrogen atmosphere. The compound **qox** was synthesized from quinuclidin-3-one hydrochloride (≥98.0%, Fluka, Honeywell Research Chemicals, Charlotte, NC USA) following published procedure [20]. Compound **1** was prepared in the reaction of **qox** with butenyl bromide, **2** was prepared with benzyl bromide, and **3**–**10** were prepared with appropriate *meta*- and *para*-substituted benzyl bromides. All halides were obtained from Sigma–Aldrich Co., St. Louis, MO, USA and used without further purification.

#### General Procedure for the Synthesis of N-quaternary 3-hydroxyiminoquinuclidinium bromides **1**–**10**

To the solution of 3-hydroxyquinuclidine (1 mmol) in minimal volume of dry tetrahydrofuran equimolar amount of the appropriate quaternization reagent (butenyl bromide benzyl bromide, *para*- and *meta*-nitrobenzyl bromide, *para*- and *meta*-chlorobenzyl bromide, *para*- and *meta*-methylbenzyl bromide, and *para*- and *meta*-bromobenzyl bromide each) was added at room temperature. The reaction mixture was refluxed overnight under nitrogen atmosphere to obtain a solid product which was washed several times with dry diethyl ether. Digestion gave appropriate quaternary compounds as white solids.

*N-but-3-enyl-3-hydroxyquinuclidinium bromide* (**1**). Yield: 62%; mp: 211.5–212.1 °C; IR (KBr) $\tilde{\nu }$/cm^−1^: 3319–2686, 1643, 1463, 1423, 1085, 838, 700. ^1^H NMR (400 MHz, DMSO-*d*_6_) *δ*/ppm: 1.89–2.14 (m, 4H, H8, H5), 2.48–2.56 (m, 2H, H10), 2.79–2.82 (m, 1H, H4), 3.37–3.66 (m, 8H, H6, H7, H9), 4.36 (s, 2H, H2), 5.13–5.26 (m, 2H, H12), 5.72–5.83 (m, 1H, H11), 11.15 (s, 1H, C=N–OH). ^13^C NMR (101 MHz, DMSO-*d*_6_) *δ*/ppm: 23.53 (C5, C8), 26.48 (C10), 26.95 (C4), 54.73 (C6, C7), 57.73 (C2), 62.48 (C9), 118.86 (C12), 133.62 (C11), 151.62 (C3). CHN analysis/%: Anal. calcd. for C_11_H_19_BrN_2_O: C 48.01; H 6.96; Br 29.04; N 10.18; O 5.81. Found: C 48.14; H 6.95; N 10.20.

*N-benzyl-3-hydroxyiminoquinuclidinium bromide* (**2**). Yield: 85%; mp: 219.7–222.6 °C; IR (KBr) $\tilde{\nu }$/cm^−1^: 3390–2689, 1673, 1460, 1419, 1063, 944, 770, 713. ^1^H NMR (400 MHz, DMSO-*d*_6_) *δ*/ppm: 1.88–2.15 (m, 4H, H5, H8), 2.76–2.81 (m, 1H, H4), 3.49–3.66 (m, 4H, H6, H7), 4.32 (m, 2H, H2), 4.66–4.69 (m, 2H, Ar-CH_2_), 7.54–7.57 (m, 5H, Ar), 11.14 (s, 1H C=N–OH). ^13^C NMR (101 MHz, DMSO-*d*_6_) *δ*/ppm: 23.49 (C5, C8), 27.47 (C4), 54.55 (C6, C7), 57.31 (C2), 66.64 (C9), 127.81 (C10), 129.55; 130.79; 133.54; (C11-C15), 151.41 (C3). CHN analysis/%: Anal. calcd. for C_14_H_19_BrN_2_O: C 54.03, H 6.15, Br 25.67, N 9.00, O 5.14. Found: C 53.88, H 6.16, N 9.02.

*N-(*para*-nitrobenzyl)-3-hydroxyiminoquinuclidinium bromide* (**3**). Yield: 91%; mp: 232.5–233.2 °C; IR (KBr) $\tilde{\nu }$/cm^−1^: 3214–2846, 1608, 1524, 1353, 958, 856, 706. ^1^H NMR (400 MHz, DMSO-*d*_6_) *δ*/ppm: 1.88–2.15 (m, 4H, H5, H8), 2.76–2.81 (m, 1H H4), 3.49–3.66 (m, 4H, H6, H7), 4.37 (m, 2H, H2), 4.83 (s, 2H, Ar-CH_2_), 7.84–7.89 (m, 2H, H11, H15), 8.36–8.41 (m, 2H, H12, H14), 11.16 (s, 1H C=N–OH). ^13^C NMR (101 MHz, DMSO-*d*_6_) *δ*/ppm: 23.52 (C5, C8), 27.33 (C4), 54.87 (C6, C7), 57.51 (C2), 65.19 (C9), 134.89 (C10), 124.40 (C11, C15), 135.14 (C12,C14), 149.12 (C13), 151.21 (C3). CHN analysis/%: Anal. calcd. for C_14_H_18_BrN_3_O_3_: C 47.20, H 5.09, Br 22.43, N 11.80, O 13.47. Found: C 47.10, H 5.08, N 11.81.

*N-(*meta*-nitrobenzyl)-3-hydroxyiminoquinuclidinium bromide* (**4**). Yield: 81%; mp: 261–262.6 °C; IR (KBr) $\tilde{\nu }$/cm^−1^: 3377–2846, 1670, 1618, 1531, 1353, 945, 707. ^1^H NMR (400 MHz, DMSO-*d*_6_) *δ*/ppm: 1.88–2.15 (m, 4H, H5, H8), 2.76–2.81 (m, 1H H4), 3.49–3.66 (m, 4H, H6, H7), 4.32 (m, 2H, H2), 4.66–4.69 (m, 2H, Ar–CH_2_), 7.80–7.89 (m, 1H, H14), 8.00–8.06 (m, 1H, H15), 8.38–8.42 (m, 1H, H13), 8.45–8.47 (m, 1H, H11), 11.16 (s, 1H C=N–OH). ^13^C NMR (101 MHz, DMSO-*d*_6_) *δ*/ppm: 23.52 (C5, C8), 27.41 (C4), 54.76 (C6, C7), 57.45 (C2), 65.21 (C9), 129.78 (C10), 125.60, 125.25, 131.16, 140.10 (C11–C15), 148.49 (C12), 151.23 (C3). CHN analysis/%: Anal. calcd. for C_14_H_18_BrN_3_O_3_: C 47.20, H 5.09, Br 22.43, N 11.80, O 13.47. Found: C 47.32, H 5.10, N 11.81.

*N-(*para*-chlorobenzyl)-3-hydroxyiminoquinuclidinium bromide* (**5**). Yield: 92%; mp: 239.7–240.3 °C; IR (KBr) $\tilde{\nu }$/cm^−1^: 3184–2781, 1652, 1598, 1493, 1437, 1384, 951, 883, 626. ^1^H NMR (400 MHz, DMSO-*d*_6_) *δ*/ppm: 1.88–2.15 (m, 4H, H5, H8), 2.76–2.81 (m, 1H H4), 3.49–3.63 (m, 4H, H6, H7), 4.32 (m, 2H, H2), 4.68 (s, 2H, Ar–CH_2_), 7.56–7.65 (m, 4H, H11, H12, H14, H15), 11.14 (s, 1H C=N–OH). ^13^C NMR (101 MHz, DMSO-*d*_6_) *δ*/ppm: 23.49 (C5, C8), 27.44 (C4), 54.54 (C6, C7), 57.28 (C2), 65.64 (C9), 126.80 (C10), 129.59 (C11, C15), 135.65 (C12,C14), 135.80 (C13), 151.35 (C3). CHN analysis/%: Anal. calcd. for C_14_H_18_BrClN_2_O: C 48.65, H 5.25, Br 23.12, Cl 10.26, N 8.10, O 4.63. Found: C 48.52, H 5.26, N 8.12.

*N-(*meta*-chlorobenzyl)-3-hydroxyiminoquinuclidinium bromide* (**6**). Yield: 89%; mp: 239.2–241.0 °C; IR (KBr) $\tilde{\nu }$/cm^−1^: 3445–2825, 1675, 1574, 1467, 1378, 934, 796, 715. ^1^H NMR (400 MHz, DMSO-*d*_6_) *δ*/ppm: 1.88–2.15 (m, 4H, H5, H8), 2.76–2.81 (m, 1H H4), 3.49–3.66 (m, 4H, H6, H7), 4.34 (m, 2H, H2), 4.68 (s, 2H, Ar–CH_2_), 7.52–7.71 (m, 4H, H11, H13, H14, H15), 11.15 (s, 1H C=N–OH). ^13^C NMR (101 MHz, DMSO-*d*_6_) *δ*/ppm: 23.50 (C5,C8), 27.39 (C4), 54.74 (C6, C7), 57.44 (C2), 65.69 (C9), 130.11 (C10), 130.81, 131.40, 132.33, 133.13 (C11-C15), 134.01 (C12), 151.32 (C3). CHN analysis/%: Anal. calcd. for C_14_H_18_BrClN_2_O: C 48.65; H 5.25; Br 23.12; Cl 10.26; N 8.10; O 4.63. Found: C 48.53, H 5.26, N 8.08.

*N-(*para*-methylbenzyl)-3-hydroxyiminoquinuclidinium bromide* (**7**). Yield: 94%; mp: 233.4–233.9 °C; IR (KBr) $\tilde{\nu }$/cm^−1^: 2992–3228, 1674, 1611, 1513, 1460, 1376, 1064, 1029, 956, 829, 689. ^1^H NMR (400 MHz, DMSO-*d*_6_) *δ*/ppm: 1.87–2.15 (m, 4H, H5, H8), 2.36 (s, 3H, CH_3_), 2.75–2.80 (m, 1H H4), 3.46–3.62 (m, 4H, H6, H7), 4.30 (m, 2H, H2), 4.62 (s, 2H, Ar–CH_2_), 7.31–7.39 (m, 2H, H11, H15), 7.40–7.46 (m, 2H, H12, H14), 11.13 (s, 1H C=N–OH), ^13^C NMR (101 MHz, DMSO-*d*_6_) *δ*/ppm: 21.34 (CH_3_), 23.48 (C5, C8), 27.50 (C4), 54.41 (C6, C7), 57.22 (C2), 66.49 (C9), 124.79 (C10), 130.09 (C11, C15), 133.41 (C12,C14), 140.50 (C13), 151.44 (C3). CHN analysis/%: Anal. calcd. for C_15_H_21_BrN_2_O: C 55.39; H 6.51, Br 24.57, N 8.61, O 4.92. Found: C 55.49, H 6.52, N 8.60.

*N-(*meta*-methylbenzyl)-3-hydroxyiminoquinuclidinium bromide* (**8**). Yield: 91%; mp: 218.6–219.4 °C; IR (KBr) $\tilde{\nu }$/cm^−1^: 3511, 3433–2758, 1671, 1659, 1466, 1418, 1381, 940, 887, 797, 711. ^1^H NMR (400 MHz, DMSO-*d*_6_) *δ*/ppm: 1.88–2.15 (m, 4H, H5, H8), 2.76–2.81 (m, 1H H4), 3.50–3.59 (m, 4H, H6, H7), 4.32 (m, 2H, H2), 4.61 (s, 2H, Ar–CH_2_), 7.32–7.45 (m, 4H, H11, H13, H14, H15), 11.14 (s, 1H C=N–OH), ^13^C NMR (101 MHz, DMSO-*d*_6_) *δ*/ppm: 21.40 (CH_3_), 23.49 (C5,C8), 27.46 (C4), 54.53 (C6, C7), 57.43 (C2), 66.74 (C9), 127.70 (C10), 129.42, 130.64, 131.40, 133.95 (C11-C15), 138.85 (C12), 151.44 (C3). CHN analysis/%: Anal. calcd. for C_15_H_21_BrN_2_O: C 55.39, H 6.51, Br 24.57, N 8.61, O 4.92. Found: C 55.43, H 6.50, N 8.59.

*N-(*para*-bromobenzyl)-3-hydroxyiminoquinuclidinium bromide* (**9**). Yield: 95%; mp: 234.9–235.7 °C; IR (KBr) $\tilde{\nu }$/cm^−1^: 3346–2840, 1656, 1630, 1588, 1460, 1376, 1072, 945, 842. ^1^H NMR (400 MHz, DMSO-*d*_6_) *δ*/ppm: 1.89–2.14 (m, 4H, H5, H8), 2.76–2.81 (m, 1H H4), 3.49–3.65 (m, 4H, H6, H7), 4.31 (m, 2H, H2), 4.66 (s, 2H, Ar–CH_2_), 7.48–7.53 (m, 2H, H11, H15), 7.73–8.79 (m, 2H, H12, H14), 11.14 (s, 1H C=N–OH), ^13^C NMR (101 MHz, DMSO-*d*_6_) *δ*/ppm: 23.49 (C5, C8), 27.43 (C4), 54.55 (C6, C7), 57.29 (C2), 65.74 (C9), 124.67 (C10), 127.13 (C13), 132.54 (C11,C15), 135.60 (C12,C14), 151.34 (C3). CHN analysis/%: Anal. calcd. for C_14_H_18_Br_2_N_2_O: C 43.10, H 4.65, Br 40.96, N 7.18, O 4.10. Found: C 43.99, H 4.64, N 7.20.

*N-(*meta*-bromobenzyl)-3-hydroxyiminoquinuclidinium bromide* (**10**). Yield: 89%; mp: 221.7–222.4 °C; IR (KBr) $\tilde{\nu }$/cm^−1^: 3481, 3360–2801, 1673, 1569, 1431, 1377, 1215, 1064, 943, 719, 711. ^1^H NMR (400 MHz, DMSO-*d*_6_) *δ*/ppm: 1.89–2.15 (m, 4H, H5, H8), 2.78–2.81 (m, 1H H4), 3.50–3.63 (m, 4H, H6, H7), 4.34 (s, 2H, H2), 4.67 (s, 2H, Ar–CH_2_), 7.48–7.54 (m, 1H, H14), 7.56–7.59 (m, 1H, H15), 7.75–7.78 (m, 1H, H13), 7.80–7.82 (m, 1H, H11), 11.15 (s, 1H C=N–OH), ^13^C NMR (101 MHz, DMSO-*d*_6_) *δ*/ppm: 23.51 (C5,C8), 27.40 (C4), 54.72 (C6, C7), 57.44 (C2), 65.64 (C9), 122.57 (C10), 130.38 (C12), 131.64, 132.69, 133.68, 135.95, (C11-C15), 151.32 (C3). CHN analysis/%: Anal. calcd. for C_14_H_18_Br_2_N_2_O: C 43.10, H 4.65, Br 40.96, N 7.18, O 4.10. Found: C 43.21, H 4.65, N 7.16.

### 3.2. Antimicrobial Evaluation

The tested microorganisms were obtained from the culture collection at the American Type Culture Collection (ATCC) (Rockville, MD, USA) and at the Microbiology laboratory, Department of Biology, Faculty of Natural Science, University of Split, Croatia (FNSST). The assayed collection included gram-positive bacteria *Bacillus cereus* (ATCC 11778), *Enterococcus faecalis* (ATCC 29212), and *Staphylococcus aureus* (ATCC 25923) and three gram-negative ampicillin-resistant environmental bacterial strains *Escherichia coli* (FNSST 111), *Klebsiella pneumoniae* (FNSST 014), and *Pseudomonas aeruginosa* (FNSST 982). All gram-negative strains were resistant to first- and second-generation cephalosporins, penicillins, quinolones, tetracyclines, gentamicin, and trimethoprim as determined previously [24]. Bacterial strains were cultured overnight at 37 °C in tryptic soy broth (TSB) to achieve optical densities corresponding to 10^6^ colony forming units (cfu/mL) for bacteria. All strains were grown in lysogenic broth (LB) media while being shaken at 150 rpm for 12 h at 37 °C. Part of the bacterial suspension (100 µL) was transferred to a vial containing 5 mL of LB media and incubated for 2 h at 37 °C to get mid logarithmic phase growth of bacteria. Finally, the bacterial suspension was diluted using a phosphate buffer saline (PBS, pH 7.2) to achieve 10^5^ bacteria/mL.

#### 3.2.1. Disc Diffusion Assay

In order to investigate the antimicrobial activities of the synthesized *N*-alkyl derivatives of quinucline-3-oximes, a disc diffusion assay was employed according to the Clinical and Laboratory Standards Institute (CLSI) guidelines [25]. Briefly, 100 µL of suspension containing 10^5^ colony-forming units (cfu/mL) of bacterial cells was spread on a Mueller Hinton agar (Becton Dickinson, Sparks, MD). The stock solutions of quaternary salts were prepared by dissolving in sterile distilled water to a final concentration of 10 mg/mL. The sterile filter discs (6 mm) were individually loaded with 25 µL of the stock solution, equivalent to a final concentration of 250 µg/disc of synthesized compounds, and then placed on the nutrient agar that had been previously inoculated with the target microbial strains. The plates were incubated for 24 h at 37 °C for the bacterial strain. Antibacterial activity was assessed by measuring the diameter of the inhibition zone in millimetres, including disc diameter for the test isolates. Samples were assayed in triplicate for each condition, and the diameters of inhibition zones were presented as mean values.

#### 3.2.2. Minimum Inhibitory Concentration Assay

In addition, antimicrobial activities of the synthesized compounds were tested by a broth microdilution assay in 96-well microtitre plates. The standard two-fold serial microdilution assay described by the Clinical and Laboratory Standards Institute [24] was performed for the assessment of the minimum inhibitory concentrations (MICs). Bacteria were grown overnight in Mueller–Hinton broth (MHB) at 37 °C to the stationary phase. The microbial cultures were diluted in fresh MHB to a final concentration of 10^5^ cfu/mL for bacteria. The tested compounds dissolved in sterile distilled water to the highest concentration of 10 mg/mL. The stock solution and reference drugs were then serially two-fold diluted to obtain concentrations ranging from 0.06–1024 µg/mL in sterile plates containing Mueller Hinton broth (MHB) for bacterial. Serial dilutions of the compounds were added to the microtiter plates in a volume of 100 µL. Each well was additionally inoculated with 100 µL of inoculums of the target microorganism and incubated at 37 °C for 18–24 h. The MIC value was determined as the lowest concentration of the sample at which the tested microorganisms did not demonstrate any visible growth after incubation. As an indicator of bacterial growth, 50 μL of 0.2 mg/mL *p*-iodonitrotetrazolium chloride (INT; Sigma-Aldrich Co. Ltd., Poole, UK) was added to the wells and incubated at 37 °C for 30 min. Following addition of INT and incubation, the MIC was determined as the lowest sample concentration at which no pink color appeared. Cefotaxime and gentamicin were used as positive controls.

### 3.3. MTT Assay

In order to investigate the effect of the compounds on normal human cells, two normal human cell lines were used, HMEC-1, human dermal microvascular endothelium cell line, and HaCaT, human keratinocyte cell line. Both cell lines were cultured in Dulbecco’s modified Eagle medium (DMEM) supplemented with 10% fetal calf serum (FCS). Cells were cultivated on 37 °C in 5% CO_2_ humidified atmosphere.

For the cell viability screening assay, all compounds were used at concentrations 1 µM, 10 µM, and 100 µM. Then, the viability assay was repeated with the compounds that showed antimicrobial activity with a wider concentration range.

Cells were seeded at 10^4^ cells/well left for 24 h to attach. The next day cells were treated, and all compounds were used at concentrations of 1 µM, 10 µM, and 100 µM. For the compounds that showed antimicrobial activity, a wider range of drug concentrations was used to calculate IC_50_.

In order to perform proper controls, the compounds were dived into two groups, water and DMSO soluble. DMSO controls were used at the equivalent concentration present in compound dilutions. Additional controls of the assay were performed with the same experimental scheme with H_2_O_2_ and kanamycin as cytotoxic and known antibiotics with serial double dilutions starting from 400 µM and 1600 µg/mL, respectively (Figure 4). After 24 h, cell viability was determined by an MTT-based assay, EZ4U, following the manufacturer’s recommendations (Biomedica, Vienna, Austria). The assay is based on the reduction of the colorless dye in mitochondria of the living cell to the yellow, water-soluble derivative. Briefly, cells were incubated with the MTT dye for an hour, and the absorbance was measured on a plate reader at 450 nm with reference wavelength at 620 nm (Easy-Reader 400 FW; SLT-Lab Instruments, GmbH, Grödig, Austria).

#### ROS

Cells were seeded at 10^4^ cells/well left for 24 h to attach. The next day, cells were incubated with 2,7-dichlorodihydrofluorescein diacetate (DCFH-DA, Fluka, Honeywell Research Chemicals, Charlotte, NC, USA) for 60 minutes. This nonfluorescent dye is deacetylized inside the cells and, after oxidation by intracellular ROS, turns fluorescent. After removing the excess dye, cells were washed once and were treated with the compounds in a range of concentrations. Additional controls of the assay were performed with the same experimental scheme with H_2_O_2_ and kanamycin as representative of ROS and known antibiotics with serial double dilutions starting from 400 µM and 1600 µg/mL, respectively (Figure 5). Fluorescence intensity was measured after 1 h on spectrofluorimeter Varian Cary Eclipse (λ_ex_ = 500 nm, λ_em_ = 529 nm).

### 3.4. Antioxidant Measurements

To assess the effects of the compounds on the cellular antioxidative system, cells were seeded at 0.5 × 10^6^ cell/well and treated with compounds **qox**, **6**, **9**, and **10** at concentrations of 5 µM and 200 µM. As controls, cells were grown only in DMEM with 10% FCS and with the addition of DMSO in equivalent concentrations to ones present in compound dilutions. Cells were harvested by trypsinization 24 h after treatment, and dry pellets were stored at −80 °C until analysis. For analysis, cells were lysed in PBS by four freeze/thaw cycles, and the debris was removed by centrifugation at 14,000 g for 15 min. The catalase activity was measured by a modified method of Goth [26,27]. For catalase activity assay, 40 μL of supernatant was mixed with 65-mM H_2_O_2_ for the start of the reaction. The reaction was stopped after 5 minutes by the addition of 100 μL of 200-mM ammonium molybdate and colour development was measured spectrophotometrically in a plate reader at 405 nm (Shimadzu UV-1601, Kyoto, Japan). One unit of catalase activity is defined as the amount of enzyme needed for the degradation of 1 μmol of H_2_O_2_/min at 25 °C. Catalase activity was expressed as units per milligram of proteins in cell lysate (U mg^−1^).

The intracellular GSH content was measured by modification of the protocol described by Tietze [28]. Briefly, 150 μL of samples diluted to 0.03 mg/mL protein was used for the the reaction, which started by the addition of freshly prepared reaction mix: 1.8-mM 5,5-dithio-bis-2-nitrobenzoic acid, 0.4 Units of GSH reductase, and 0.6-mM NADPH in phosphate buffer (100-mM NaH_2_PO_4_, 5-mM EDTA pH 7.4). The formation of 2-nitro-5-thiobenzoic acid was monitored spectrophotometrically in a plate reader at 405 nm (Shimadzu UV-1601, Japan). GSH concentration in cell lysates was expressed as µM of GSH per milligram of total protein (nmol mg^−1^).

### 3.5. Statistical Analysis

All experiments were performed in biological and technical triplicates. The inhibitory concentrations of 50% (IC_50_) were calculated using nonlinear regression curve fitting log(inhibitor) vs. response and variable slope with a least squares (ordinary) fit, using GraphPadPrism Version 5 software (grAphPad Corporation, San Diego, CA, USA). Statistical analyses were performed using two-way analysis of variance (ANOVA) with Bonferroni post hoc test and Student’s t-test using the same software. Values of *P* < 0.05 were considered significant.

### 3.6. Computational Methods

Conformational analysis for all compounds was performed by using the tensor decomposition of ab initio molecular dynamics trajectories using the previously published procedure [23]. Ab initio molecular dynamics were performed using on-the-fly calculations of forces in each point of the simulation and integration with the velocity Verlet algorithm. The forces were calculated using the PM7 method [29] implemented in MOPAC2016 (Stewart Computational Chemistry, Colorado Springs, CO, USA) [30]. All molecular dynamics simulations were performed using our own program qcc (T. Hrenar, Zagreb, Croatia) [31,32]. To ensure that most of the phase space relevant to the conformational space of the investigated compounds was adequately sampled, the initial temperature for the Maxwell distribution of velocities was set at 673.15 K, and this temperature was kept constant throughout simulations using a velocity scaling algorithm. The step size was 0.5 fs, and the total length of simulation was 2.5 ns (a total of 5,000,000 steps).

Tensor Decomposition: Molecular dynamics trajectories were decomposed using the principal component analysis implemented in program moonee (T. Hrenar, Zagreb, Croatia) [33,34,35]. Local maxima from corresponding probability distribution were determined and used as an initial guess for geometry optimization of structures at the B3LYP-D3/6-311++G(d,p) level of the theory. After geometry optimizations, clustering of obtained geometries produced the full conformational space of each investigated compound. All quantum-chemical calculations were performed using the Gaussian 16 program (Gaussian, Inc., Wallingford, CT, USA) [36]. Figures were plotted using graphing utility within free and open-source program Gnuplot 5.0. [37].

Potential energy surfaces: Potential energy surfaces for conformers of the lowest energy were calculated by systematic variation of torsional angles for the chemical groups attached to the nitrogen atom. For each point, an energy calculation for the structure was performed using the Gaussian 16 program package [36]. Energy values were arranged in data matrix or 2nd-order data tensors and combined for all investigated compounds, making a 3rd-order data tensor. This tensor was decomposed by using Tucker3 tensor decomposition method [38].

## 4. Conclusions

The antimicrobial profiles of presented novel *N*-alkyl derivatives of quinuclinium oximes **1**–**10** were determined. Compounds **5** and **10** (MIC values 0.25 μg/mL) demonstrated a very high potential in combating multidrug-resistant gram-negative bacterial strains, especially the emerging pathogen *Pseudomonas aeruginosa*. Both compounds showed broad spectrum antimicrobial activity against gram-positive and gram-negative bacteria. Compounds have no inhibitory effects on normal human cells growth and are suitable for further development. Continued studies on these molecules, including combinatorial therapies with conventional antibiotics and animal modeling, will enable the elucidation of the mechanisms by which these derivatives provide a potent antimicrobial effect. Regression model between the biological activity and potential energy surfaces was established using the tensor reduction techniques. The high *R*^2^ value of the established model confirms the capabilities of the model to describe the biological activity of investigated compounds in relation to their potential energy surfaces.

## Figures and Tables

**Table 1:** Antimicrobial activity of 3-hydroxyiminoquinuclidine (**qox**) and its quaternary salts (**1**–**10**) by disc diffusion assay.

Comp.	Diameters of the Inhibition Zone (mm)					
	Gram-Positive Bacteria			Gram-Negative Bacteria		
	*Bacillus cereus*	*Enterococcus faecalis*	*Staphylococcus aureus*	*Escherichia coli*	*Klebsiella pneumoniae*	*Pseudomonas aeruginosa*
**qox**	8.2 ± 0.9	7.7 ± 1.4	10.4 ± 1.7	8.6 ± 1.9	7.6 ± 1.1	10.8 ± 1.4
**1**	9.9 ± 1.4	6.2 ± 1.3	9.3 ± 0.8	9.6 ± 1.1	8.4 ± 1.1	12.1 ± 1.8
**2**	12.1 ± 1.8	14.2 ± 2.1	15.7 ± 1.7	14.8 ± 1.2	16.1 ± 0.9	19.2 ± 1.4
**3**	10.4 ± 1.5	12.7 ± 1.4	13.5 ± 1.7	15.1 ± 1.2	13.7 ± 1.8	16.3 ± 1.5
**4**	16.1 ± 0.4	15.4 ± 1.2	18.1 ± 1.1	17.7 ± 0.9	14.7 ± 0.4	18.7 ± 0.9
**5**	18.2 ± 1.2	19.4 ± 1.8	20.8 ± 1.1	20.8 ± 2.1	19.8 ± 0.4	24.8 ± 1.7
**6**	16.8 ± 0.8	17.4 ± 1.7	17.2 ± 0.3	19.1 ± 1.5	18.1 ± 1.8	20.1 ± 2.1
**7**	10.8 ± 1.7	6.4 ± 2.1	9.5 ± 1.3	8.1 ± 1.2	7.5 ± 1.4	11.0 ± 0.8
**8**	7.8 ± 0.3	9.6 ± 1.2	11.4 ± 0.7	6.6 ± 1.1	8.6 ± 1.1	10.6 ± 1.1
**9**	23.5 ± 1.3	17.3 ± 2.4	27.2 ± 1.3	22.7 ± 0.8	24.7 ± 1.3	27.6 ± 1.7
**10**	24.0 ± 1.2	23.0 ± 1.4	21.0 ± 0.8	22.4 ± 0.9	25.1 ± 0.6	29.8 ± 1.3
**Gen**	18.2 ± 0.7	13.2 ± 0.6	23.9 ± 0.4	11.5 ± 0.9	16.9 ± 0.3	9.4 ± 0.6

**Table 2:** In vitro antibacterial activities for compounds **1**–**10** expressed as minimum inhibitory concentrations (MIC; µg/mL) ^a,b^.

Comp.	MIC (µg/mL)					
	Gram-Positive Bacteria			Gram-Negative Bacteria		
	*B. cereus*	*E. faecalis*	*S. aureus*	*E. coli*	*K. pneumoniae*	*P. aeruginosa*
**1**	128.00	256.00	128.00	128.00	256.00	128.00
**2**	64.00	64.00	32.00	16.00	16.00	8.00
**3**	64.00	32.00	32.00	16.00	64.00	32.00
**4**	32.00	16.00	16.00	32.00	32.00	16.00
**5**	4.00	2.00	1.00	1.00	2.00	0.25
**6**	8.00	4.00	4.00	4.00	8.00	2.00
**7**	64.00	128.00	64.00	128.00	256.00	32.00
**8**	128.00	64.00	64.00	256.00	128.00	128.00
**9**	1.00	8.00	12.50	25.00	50.00	0.50
**10**	1.00	4.00	2.00	1.00	0.50	0.25
**Gentamicin**	4.00	4.00	1.00	32.00	8.00	64.00
**Cefotaxime**	0.25	0.50	0.50	0.50	0.50	16.00

^a^ Minimum inhibitory concentrations were determined by the micro broth dilution method for microdilution plates. ^b^ Gentamicin and cefotaxime were used as the positive controls.

## References

[1] Fauci A.S. (2001). Infectious Diseases: Considerations for the 21st Century. Clin. Infect. Dis..

[2] Boucher H.W., Talbot G.H., Bradley J.S., Edwards J.E., Gilbert D., Rice L.B., Scheld M., Spellberg B., Bartlett J. (2009). Bad bugs, no drugs: No ESKAPE! An update from the Infectious Diseases Society of America. Clin. Infect. Dis..

[3] Pendleton J.N., Gorman S.P., Gilmore B.F. (2013). Clinical relevance of the ESKAPE pathogens. Expert. Rev. Anti. Infect. Ther..

[4] World Health Organization (2014). Antimicrobial Resistance: Global Report on Surveillance.

[5] Gomtsyan A. (2012). Heterocycles in drugs and drug discovery. Chem. Heterocycl. Compd..

[6] Mittal A. (2009). Synthetic Nitroimidazoles: Biological Activities and Mutagenicity Relationships. Sci. Pharm..

[7] Nagalakshmi G. (2008). Synthesis, antimicrobial and antiinflammatory activity of 2,5-disubstituted-1,3,4-oxadiazoles. Indian J. Pharm. Sci..

[8] Joule J.A., Mills K. (2000). Heterocyclic Chemistry.

[9] Nekrasov D.D. (2001). Biological Activity of 5-and 6-Membered Azaheterocycles and Their Synthesis from 5-Aryl-2,3-Dihydrofuran-2,3-diones. Chem. Heterocycl. Compd..

[10] Sperry J.B., Wright D.L. (2005). Furans, thiophenes and related heterocycles in drug discovery. Curr. Opin. Drug Discovery Dev..

[11] Polshettiwar V., Varma R.S. (2008). Greener and expeditious synthesis of bioactive heterocycles using microwave irradiation. Pure Appl. Chem..

[12] Katritzky A.R. (1992). Heterocyclic chemistry: An academic subject of immense industrial importance. Chem. Heterocycl. Compd..

[13] Song C.E. (2009). An Overview of Cinchona Alkaloids in Chemistry.

[14] Gralla R., Lichinitser M., Van der Vegt S., Sleeboom H., Mezger J., Peschel C., Tonini G., Labianca R., Macciocchi A., Aapro M. (2003). Palonosetron improves prevention of chemotherapy-induced nausea and vomiting following moderately emetogenic chemotherapy: Results of a double-blind randomized phase III trial comparing single doses of palonosetron with ondansetron. Ann. Oncol..

[15] Bazina L., Maravić A., Krce L., Soldo B., Odžak R., Bučević Popović V., Aviani I., Primožič I., Šprung M. (2019). Discovery of novel quaternary ammonium compounds based on quinuclidin-3-ol as new potential antimicrobial candidates. Eur. J. Med. Chem..

[16] Odžak R., Šprung M., Soldo B., Skočibušić M., Gudelj M., Muić A., Primožič I. (2017). Quaternary salts derived from 3-substituted quinuclidine as potential antioxidative and antimicrobial agents. Open Chem..

[17] Skočibušić M., Odžak R., Štefanić Z., Križić I., Krišto L., Jović O., Hrenar T., Primožič I., Jurašin D. (2016). Structure-Property Relationship of Quinuclidinium Surfactants—Towards Multifunctional Biologically Active Molecules. Colloids Surf. B Biointerfaces.

[18] Gerba C.P. (2015). Quaternary Ammonium Biocides: Efficacy in Application. Appl. Environ. Microbiol..

[19] Grob C.A., Renk E. (1954). 3-Chinuclidincarbonsaure. Helv. Chim. Acta.

[20] Sternbach L.H., Kaiser S. (1952). Antispasmodics. I. Bicyclic Basic Alcohols. J. Am. Chem. Soc..

[21] World Health Organization (2017). Critically Important Antimicrobials for Human Medicine: Ranking of Antimicrobial Agents for Risk Management of Antimicrobial Resistance Due to Non-Human Use.

[22] Tam V.H., Kabbara S., Vo G., Schilling A.N., Coyle E.A. (2006). Comparative pharmacodynamics of gentamicin against Staphylococcus aureus and Pseudomonas aeruginosa. Antimicrob. Agents Chemother..

[23] Hrenar T., Primožič I., Fijan D., Majerić Elenkov M. (2017). Conformational analysis of spiro-epoxides by principal component analysis of molecular dynamics trajectories. Phys. Chem. Chem. Phys..

[24] Maravić A., Skočibušić M., Cvjetan S., Šamanić I., Fredotović Ž., Puizina J. (2015). Prevalence and diversity of extended-spectrum-β-lactamase-producing Enterobacteriaceae from marine beach waters. Mar. Pollut. Bull..

[25] Clinical and Laboratory Standards Institute (2008). Performance Standards for Antimicrobial Susceptibility Testing.

[26] Góth L.A. (1991). Simple method for determination of serum catalase activity and revision of reference range. Clin. Chim. Acta.

[27] Campbell M.K., Farrell S.O. (2012). Biochemistry.

[28] Tietze F. (1969). Enzymic method for quantitative determination of nanogram amounts of total and oxidized glutathione: Applications to mammalian blood and other tissues. Anal. Biochem..

[29] Stewart J.J.P. (2013). Optimization of parameters for semiempirical methods VI: More modifications to the NDDO approximations and re-optimization of parameters. J. Mol. Model..

[30] Stewart J.J.P. (2016). Stewart Computational Chemistry.

[31] Hrenar T. (2019). QCC, Quantum Chemistry Code.

[32] Primožič I., Hrenar T., Baumann K., Krišto L., Križić I., Tomić S. (2014). Mechanochemical and Conformational Study of N-heterocyclic Carbonyl-Oxime Transformations. Croat. Chem. Acta.

[33] Hrenar T. (2019). moonee, Code for Manipulation and Analysis of Multi- and Univariate Data.

[34] Jović O., Smolić T., Jurišić Z., Meić Z., Hrenar T. (2013). Chemometric Analysis of Croatian Extra Virgin Olive Oils from Central Dalmatia Region. Croat. Chem. Acta.

[35] Novak P., Kišić A., Hrenar T., Jednačak T., Miljanić S., Verbanec G. (2011). In-line reaction monitoring of entacapone synthesis by Raman spectroscopy and multivariate analysis. J. Pharm. Biomed. Anal..

[36] Frisch M.J., Trucks G.W., Schlegel H.B., Scuseria G.E., Robb M.A., Cheeseman J.R., Scalmani G., Barone V., Petersson G.A., Nakatsuji H. (2016). Gaussian 16, Revision A.03.

[37] Gnuplot 5.0. http://gnuplot.info/.

[38] Tucker L. (1966). Some mathematical notes on three-mode factor analysis. Psychometrika.

